# Editorial: Reimagining communication in a post-pandemic world: The intersection of information, media technology, and psychology

**DOI:** 10.3389/fpsyg.2023.1154044

**Published:** 2023-02-20

**Authors:** Anfan Chen, Hichang Cho, Richard Evans, Runxi Zeng

**Affiliations:** ^1^School of Journalism and Communication, The Chinese University of Hong Kong, Shatin, Hong Kong SAR, China; ^2^Department of Communications and New Media, Faculty of Arts and Social Sciences, National University of Singapore, Singapore, Singapore; ^3^Faculty of Computer Science, Dalhousie University, Halifax, NS, Canada; ^4^School of Journalism and Communication, Chongqing University, Chongqing, China

**Keywords:** COVID-19, pandemic, social media, media use, information processing, communication, psychology, China

The COVID-19 pandemic dramatically changed the nature of social interaction, creating negative impacts and challenges, but also opportunities for progressing how we communicate, as humans. Social distancing policies, lockdown measures, and mandatory quarantines accelerated technological communication. For example, Artificial Intelligence (AI) mediated communication grew at an unprecedented rate, willingly or otherwise. As part of pandemic control efforts, many activities, such as workplace meetings, education, and conferences, moved online, using social media platforms, the metaverse, and specialized programs accessed through mobile devices or laptops. As a result, digitally mediated channels became critical for information acquisition and communication across a wide spectrum of human activities in our personal and professional lives. As the scientific understanding of COVID-19 improved, pandemic restrictions loosened. However, it remains to be seen whether the pandemic communication paradigm, characterized by heavy technological mediation and reliance on non-human agents, will gradually decline, or whether the paradigm shift will become deeply entrenched with further acceleration of the dependency on technological mediation and non-human agents.

Such unprecedented reliance on technological mediation and non-human agents for information and communication is akin to a social-psychological experiment on a global scale. Much remains unknown, however, since this communication paradigm shift began almost 3 years ago. There are two main problems that require attention. First, the psychological impact of and underlying mechanisms behind the extended and extensive reliance on technological mediation and non-human agents is not yet well understood, especially regarding the influence of AI technologies. Second, the extent to which the impact of this reliance is likely to persist and influence future communication is also not well understood, especially in different national and cultural contexts. Research involving new approaches to data collection, such as big data techniques, together with more traditional data techniques, can help in providing greater insights into these two problems.

In this Research Topic, we sought to extend current understanding about how the COVID-19 pandemic has reshaped human communication, especially cognitive, psychological, and behavioral shifts in social interactions. We focused on research that explored new possibilities for interpersonal communication practices in the post-pandemic era. Eighteen manuscripts were selected for inclusion and draw intensive attention with more than 26,000 views.

## Social media use during the COVID-19 pandemic

Information technologies, such as social media platforms, offer individuals various functionality to support the maintenance, development, and sustainability of individuals and organizations. For instance, when COVID-19 emerged, the provision of resources and social support became a major concern for many.

In the article titled “*The Importance of Project Description to Charitable Crowdfunding Success: The Mediating Role of Forwarding Times*,” Lu et al. considered 205 COVID-19 charitable projects and 11,177,249 donors to assess the process by which nonprofit organizations raise funds through information descriptions about project descriptions. The results of the study indicated that understandability and a negative tone for descriptions help to improve the amounts raised. A question remains: might quarantines and economic disadvantages exacerbate social anxiety among impoverished individuals?

In the article titled “*Social Media as Online Shelter: Psychological Relief in COVID-19 Pandemic Diaries*,” Feng et al. explored how individual narratives on social media affect people's psychological health during a state of emergency using data collected from 19 interviews with Chinese diary writers. The study found that a pandemic diary could promote self-relief, public communication, emotional drive, meaning connection, and identity construction in public spaces, thus helping shape a sense of unity and belonging and facilitating the psychological reconstruction of people who are vulnerable to potential mental health crises.

In the article titled “*Online Collaborative Documents as Media Logic: The Mediatization of Risk Response in the Post-pandemic Era*,” Jiang et al. used a mixed-method design approach to examine treating online collaborative documents as a special approach to risk response in public health and natural disaster situations. The study found that the editability, accessibility, activability, and normality of technological affordances connected the functional features of a digital platform with users' potential actions.

## Benefits and drawbacks of information technology adoption

In this Research Topic, the authors also explored the drawbacks of information technology adoption. As the COVID-19 pandemic progressed, human reliance on information technology increased. For example, social media and the metaverse are now routinely being used for entertainment, learning, daily communication, and work.

In the article titled “*Personal network protects, social media harms: Evidence from two surveys during the COVID-19 pandemic*,” Ren and Yan demonstrated the increasingly critical and multifaceted role of communication technologies in affecting mental health conditions, indicating the destructive outcomes of the overuse of social media.

In the article titled “*How do Internet moms raise children? The reshaping of Chinese urban women's parenting psychology by COVID-19 online practices*,” Zhao and Ju, focusing on a special group of internet mothers, examined how they raised their children using the internet during the pandemic. The study found that social media created a new platform for information empowerment, mutual action, and ideation of motherhood for urban women formerly bound to family and parenting matters.

In the article titled “*Metaverse as a possible tool for reshaping schema modes in treating personality disorders*,” Yin et al. showed the potential role of the metaverse and virtual worlds in reshaping schema modes for treating personality disorders by reconstructing a new object world for a patient with the prescription of a psychotherapist.

In the article titled “*Psychological distance and user engagement in online exhibitions: Visualization of moiré patterns based on electroencephalography signals*,” Li J. et al. followed an Electroencephalography (EEG) signaling approach to highlight the possibility of EEG-visualization media devices in reducing the psychological distance and promotion of interpersonal communication between two participants experiencing an online exhibition.

## Social media use in the post-pandemic contexts

Social media also provides new opportunities for many as we enter a post-pandemic era, including the public, institutions, media, and governments.

In the articles titled “*The application of network agenda setting model during the COVID-19 pandemic based on latent Dirichlet allocation topic modeling*” and “*Event history analysis of the duration of online public opinions regarding major health emergencies*,” Liu K. et al.  and Liu X. et al. explored the public opinion landscape of the pandemic, as well as the dynamics of public opinion evolution.

In the articles titled “*Social media interactions between government and the public: A Chinese case study of government WeChat official accounts on information related to COVID-19*” and “*Government crisis communication innovation and its psychological intervention coupling: Based on an analysis of China's provincial COVID-19 outbreak updates*,” Shao et al. and Zhou et al. drew on the perspective of government-public relationships to focus on issues pertaining to government-public interactions and government crisis communication in an attempt to provide practical implications for crisis communication systems and public administrations during a public health crisis.

In the article titled “*Relationship Between Hardiness and Social Anxiety in Chinese Impoverished College Students During the COVID-19 Pandemic: Moderation by Perceived Social Support and Gender*,” Cheng et al. studied 673 impoverished Chinese college students and found that perceived social support moderated the effect of hardiness on social anxiety.

In the article titled “*Self-Efficacy, Proxy Efficacy, Media Literacy, and Official Media Use in COVID-19 Pandemic in China: A Moderated Mediation Model*,” Li Q. et al. explored the determinants of self-efficacy for fighting against the COVID-19 pandemic under social cognitive theory. The authors found that official media use was a negative moderator of the association between media literacy and proxy efficacy.

In the article titled “*Unpacking the effects of personality traits on algorithmic awareness: The mediating role of previous knowledge and moderating role of internet use*,” Fang and Jin revealed that previous knowledge of algorithms and internet use are mediators and moderators between personality and algorithm awareness.

In the article titled “*The mediating role of personal values between COVID-19-related posttraumatic growth and life satisfaction among Chinese college students: A two-wave longitudinal study*,” Xie et al. established that COVID-19-related posttraumatic growth is positively associated with life satisfaction, while self-transcendence and self-enhancement values partially mediate this association.

In the article titled “*Personal space increases during the COVID-19 pandemic in response to real and virtual humans*,” Holt et al. suggested that personal space boundaries were expanded during the pandemic. The authors provided an index of recovery from the psychological effects of the crisis.

In the article titled “*Exploring factors that influence COVID-19 vaccination intention in China: Media use preference, knowledge level and risk perception*,” Chen et al. examined the role of media use preference, knowledge level, and risk perception in predicting people's intentions to take a COVID-19 vaccine in the Chinese context.

Finally, in the article titled “*Influence of personality traits on online self-disclosure: Considering perceived value and degree of authenticity separately as mediator and moderator*,” Lv et al. revealed the role of personality traits in online selfdisclosure, while separately assessing the perceived value and degree of authenticity as mediator and moderator.

In summary, this Research Topic aimed to unite efforts to explore various aspects of communicative practices during and after a major crisis, although most of the studies were situated in the Chinese context. While we cannot say that this Research Topic provides a comprehensive knowledge map of post-pandemic communication practices, we hope that it will contribute to broadening the scope of conventional theoretical versions of information, media technology, and psychology.

## Author contributions

All authors listed have made a substantial, direct, and intellectual contribution to the work and approved it for publication.

